# Supplementary material for: Protective effects of klotho on palmitate-induced podocyte injury in diabetic nephropathy
Source: PLoS One. 2021 Apr 23;16(4):e0250666. doi: 10.1371/journal.pone.0250666 (PMC8064606; doi:10.1371/journal.pone.0250666)

Full unedited gels for Fig 1b

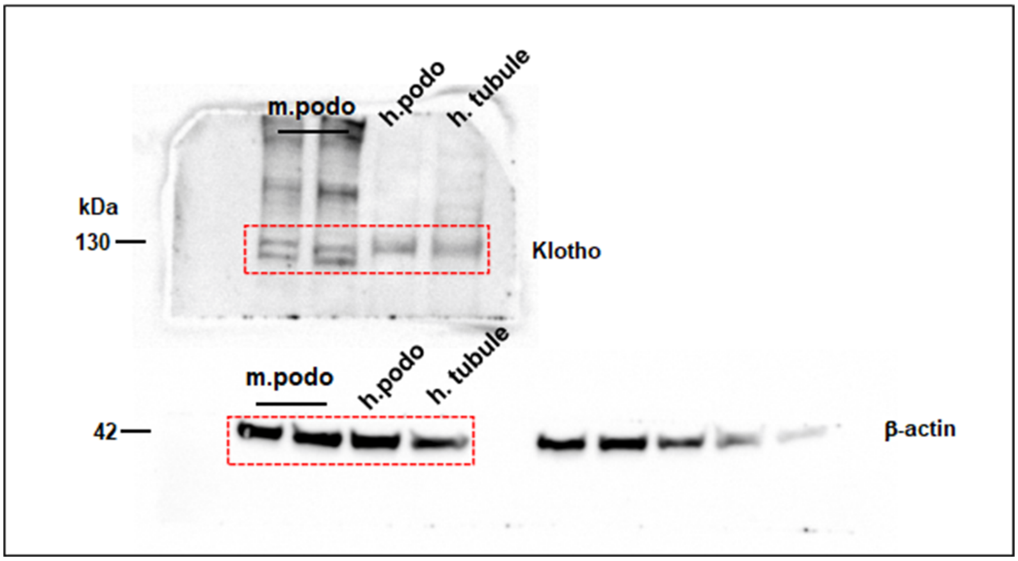

Full unedited gels for Fig 1d

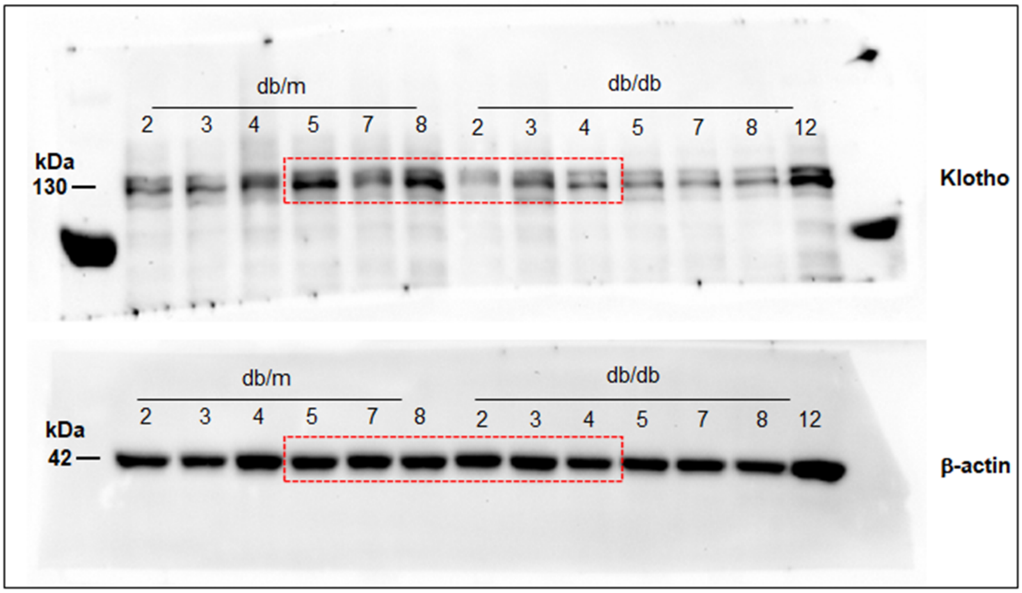

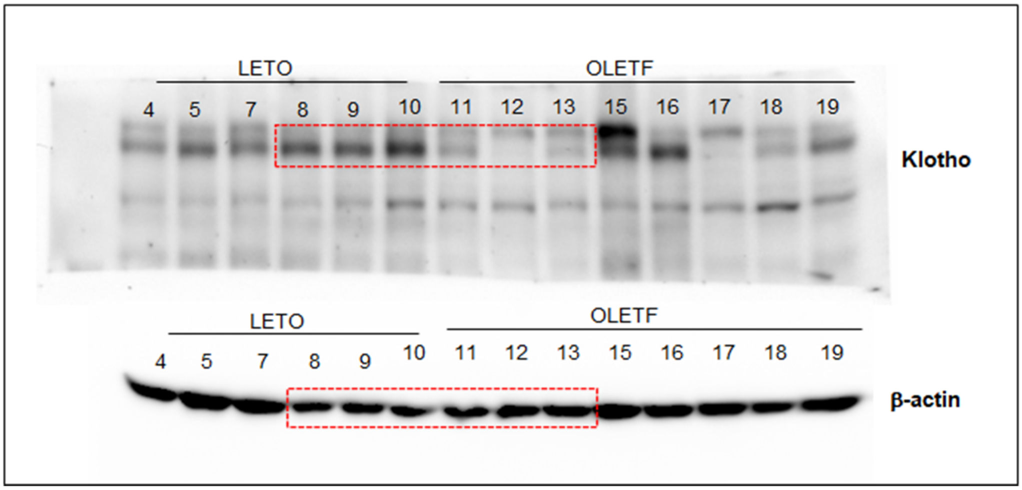

Full unedited gels for Fig 2c

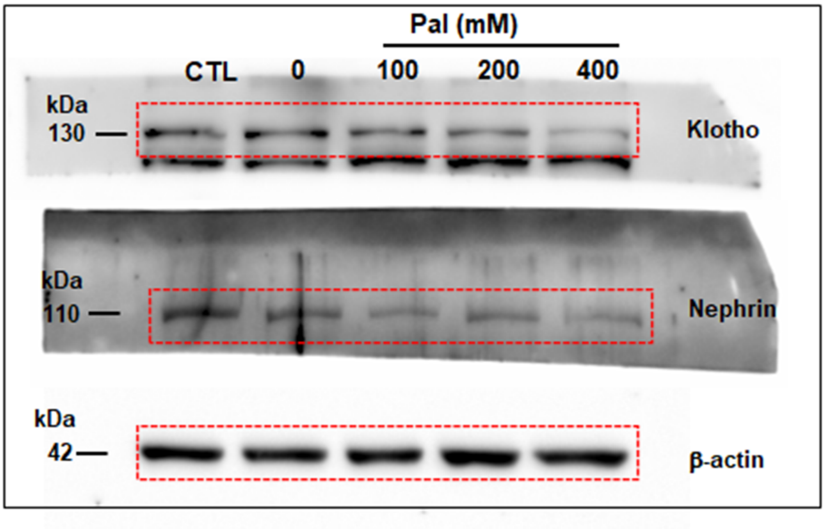

Full unedited gels for Fig 3c

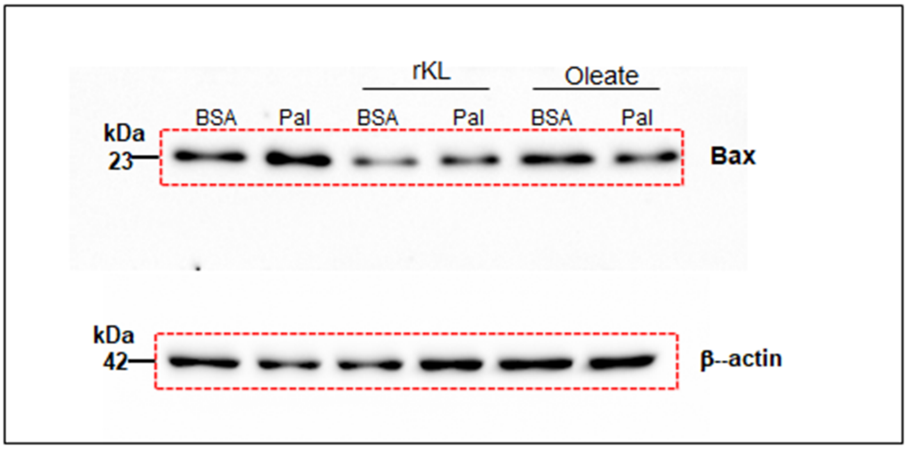

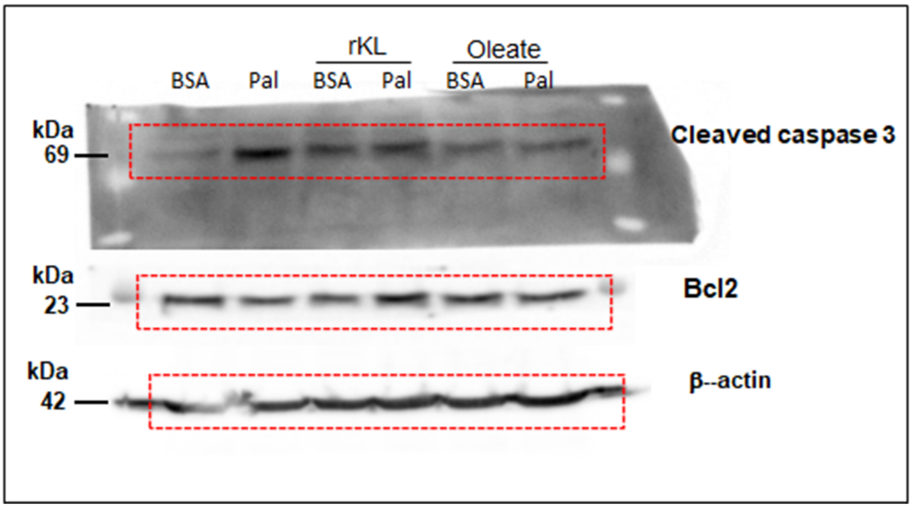

Full unedited gels for Fig 4c

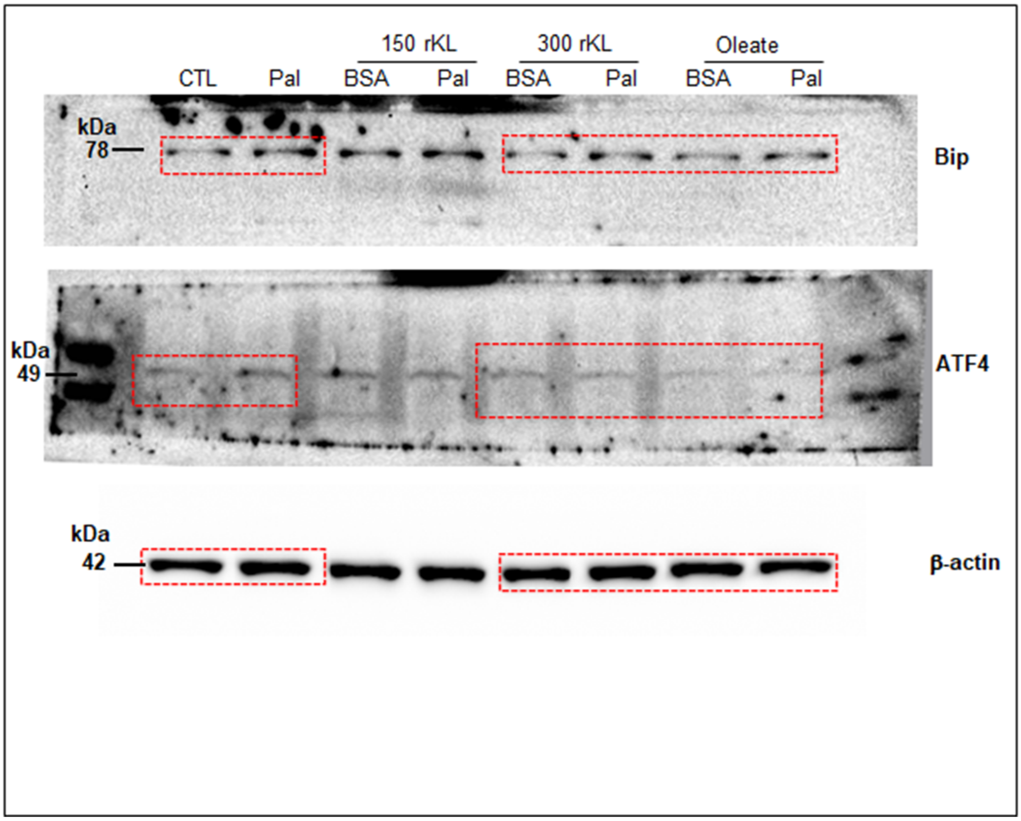

Full unedited gels for Fig 5a

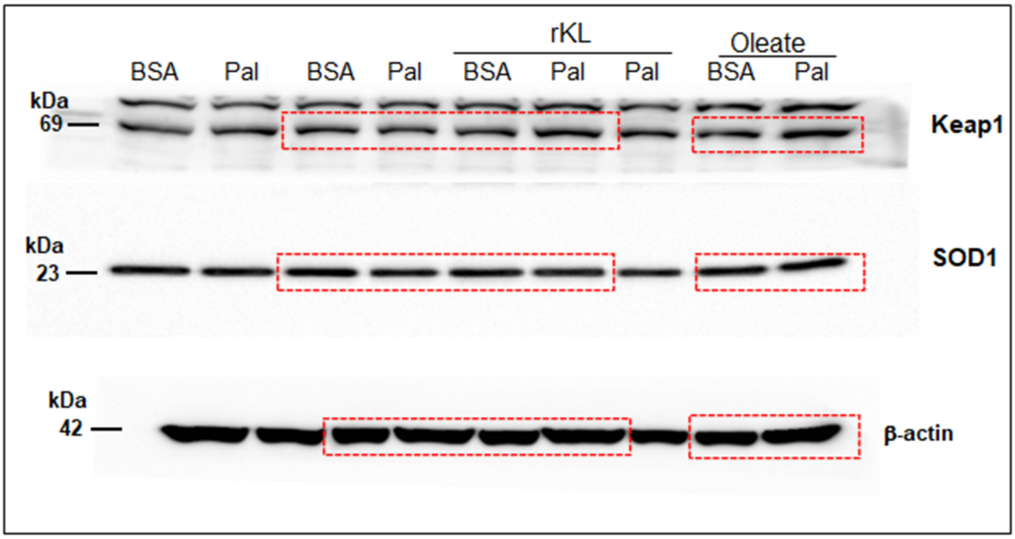

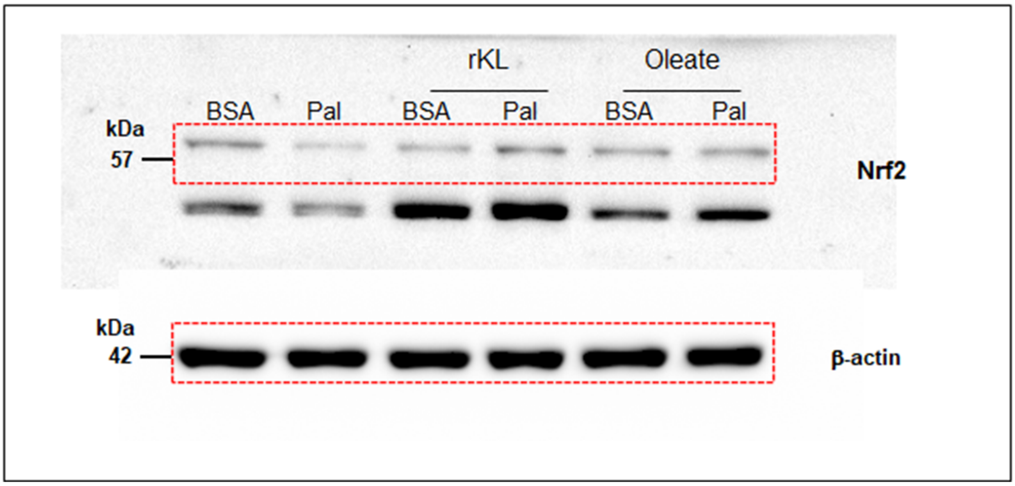

Full unedited gels for Fig 5b

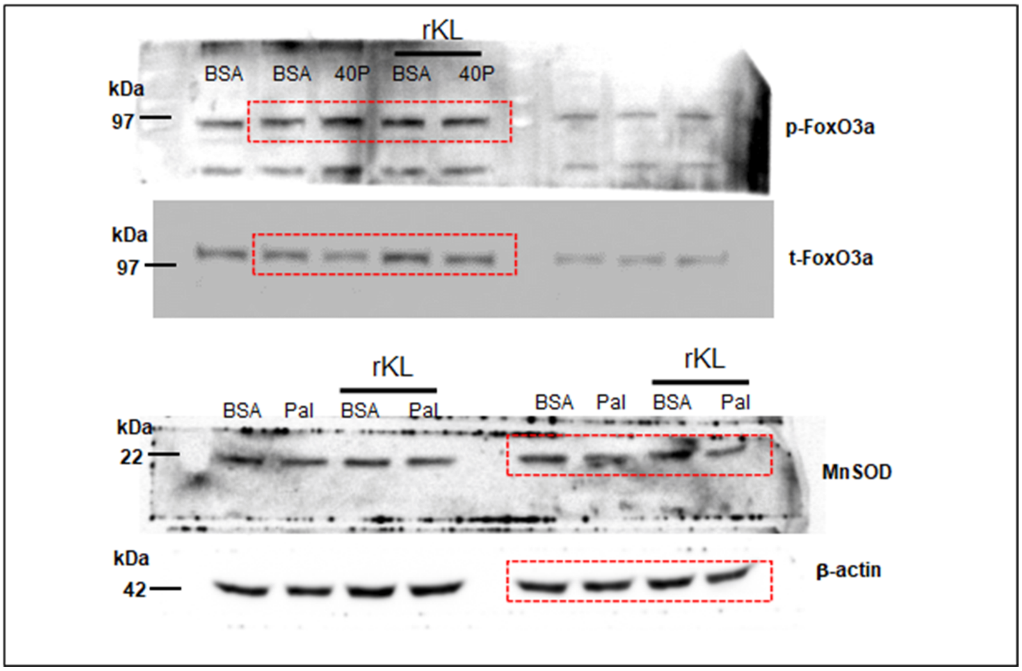

Full unedited gels for Fig 6a

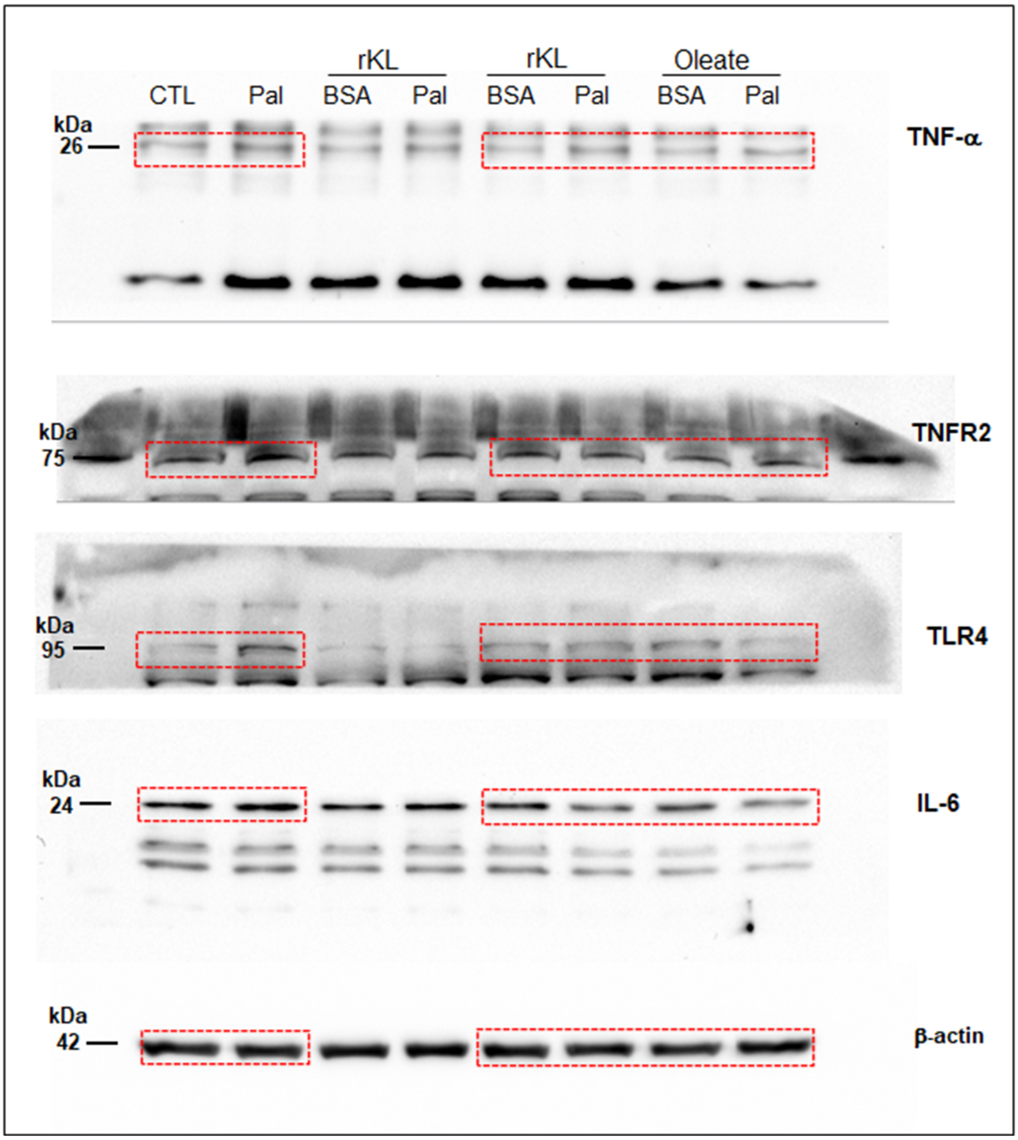

Full unedited gels for Fig 6c

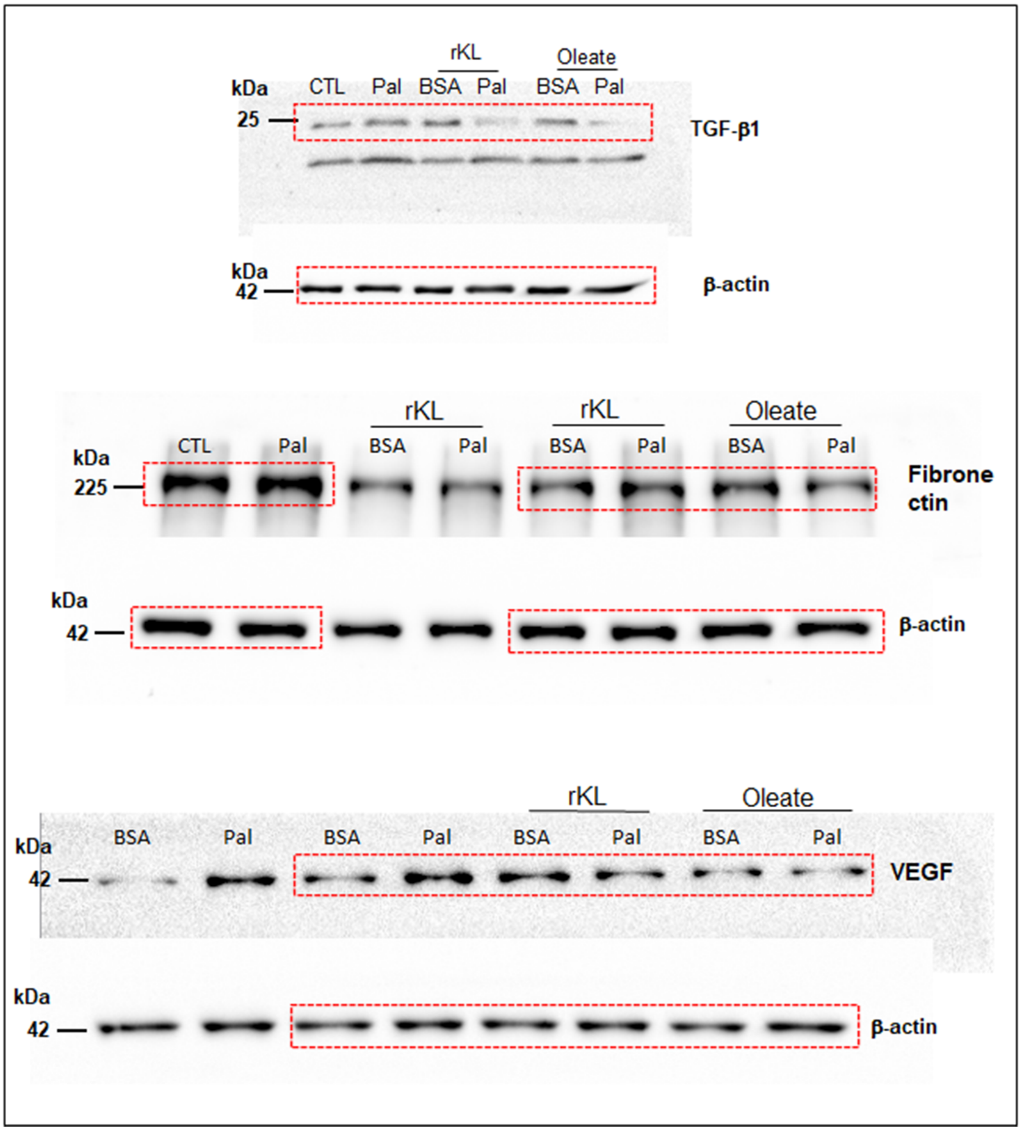

Full unedited gels for Fig 7b

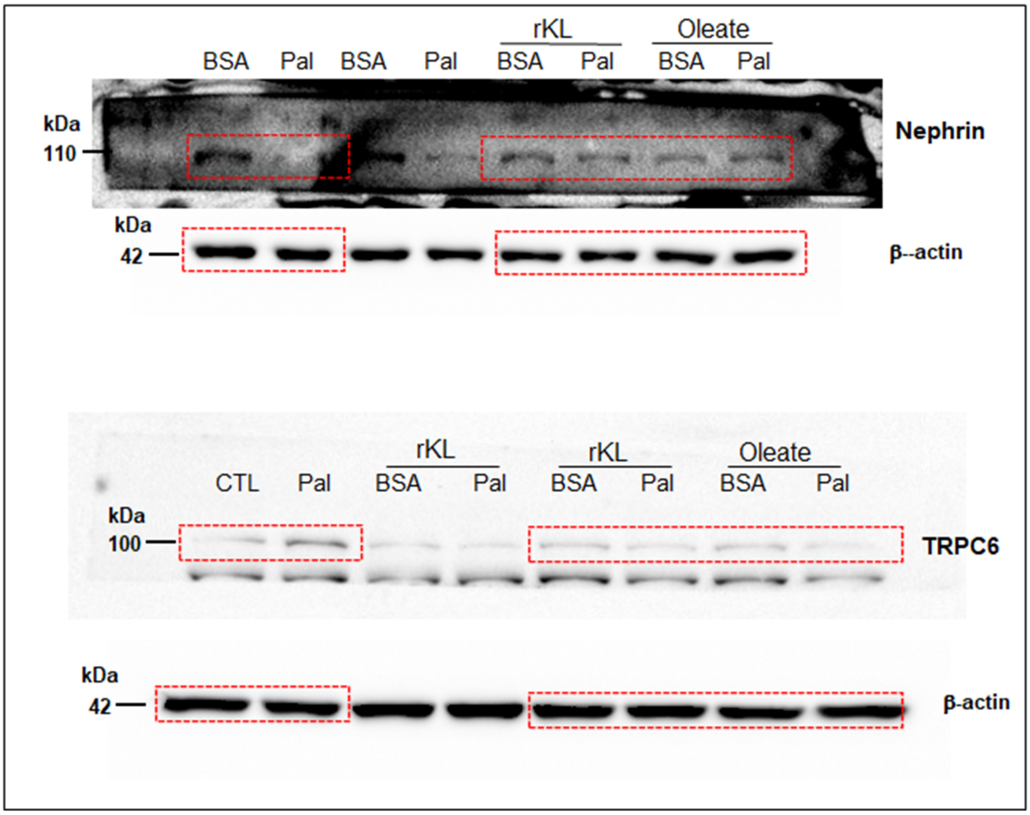

Full unedited gels for Supplementary Fig 1b

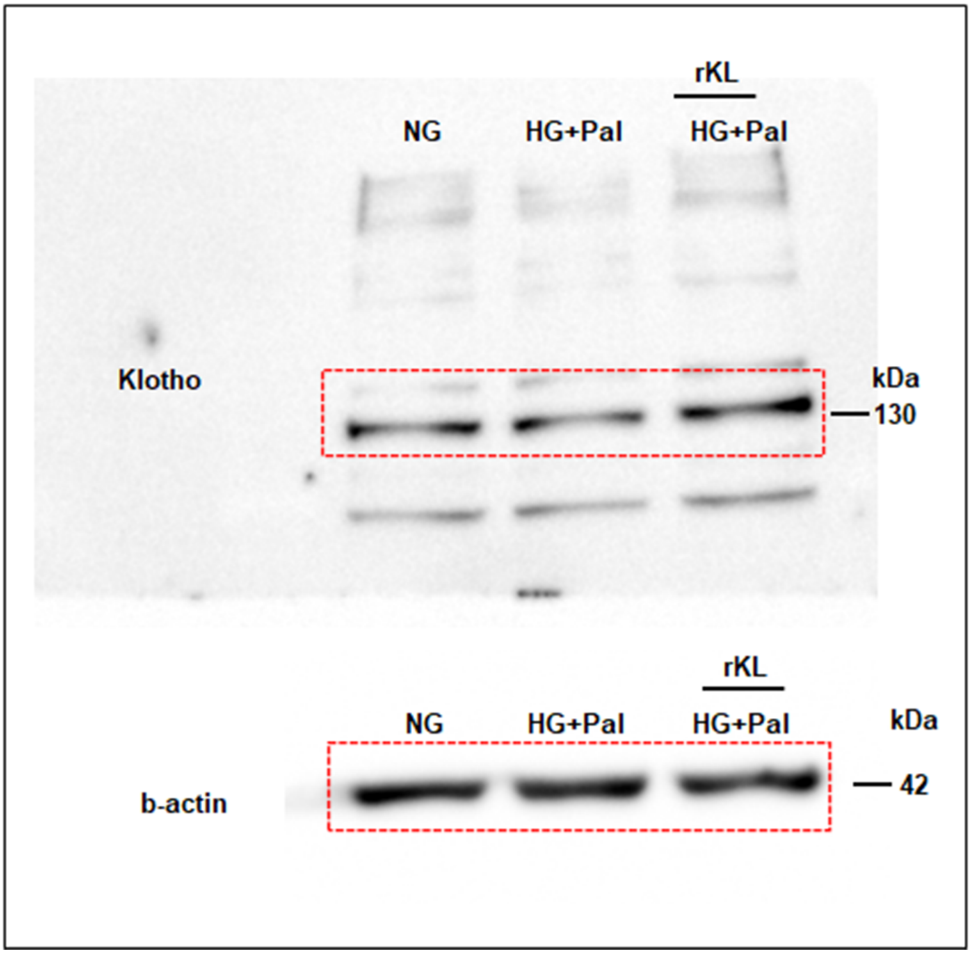

Supplement: S1 Raw images — (PDF) [file pone.0250666.s001.pdf]
