# Supplementary material for: Protective effects of klotho on palmitate-induced podocyte injury in diabetic nephropathy
Source: PLoS One. 2021 Apr 23;16(4):e0250666. doi: 10.1371/journal.pone.0250666 (PMC8064606; doi:10.1371/journal.pone.0250666)
Supplement: S1 File — (DOCX) [file pone.0250666.s002.docx]

**Protective effects of klotho on palmitate-induced podocyte injury in diabetic nephropathy**

Jeong Suk Kang^1,2^, Seung Seob Son^1^, Ji-Hye Lee^3^, Seong Woo Lee^1,4^, Ah Reum Jeong^1^, Eun Soo Lee^5^, Seung-Kuy Cha^6^, Choon Hee Chung^5^, and Eun Young Lee^1,2,4*^

^1^Department of Internal Medicine, Soonchunhyang University Cheonan Hospital, Cheonan, Korea

^2^Institute of Tissue Regeneration, College of Medicine, Soonchunhyang University, Cheonan, Korea

^3^Department of Pathology, Soonchunhyang University Cheonan Hospital, Cheonan, Korea

^4^BK21 Four project, College of Medicine, Soonchunhyang University, Cheonan, Korea

^5^Department of Internal Medicine, Yonsei University Wonju College of Medicine, Wonju, Korea

^6^Department of Physiology, Yonsei University Wonju College of Medicine, Wonju, Korea

^*^Corresponding author

E-mail: [eylee@sch.ac.kr](mailto:eylee@sch.ac.kr) (EYL)

**S1 Table. Clinical characteristics of patients with diabetic nephropathy.**

**
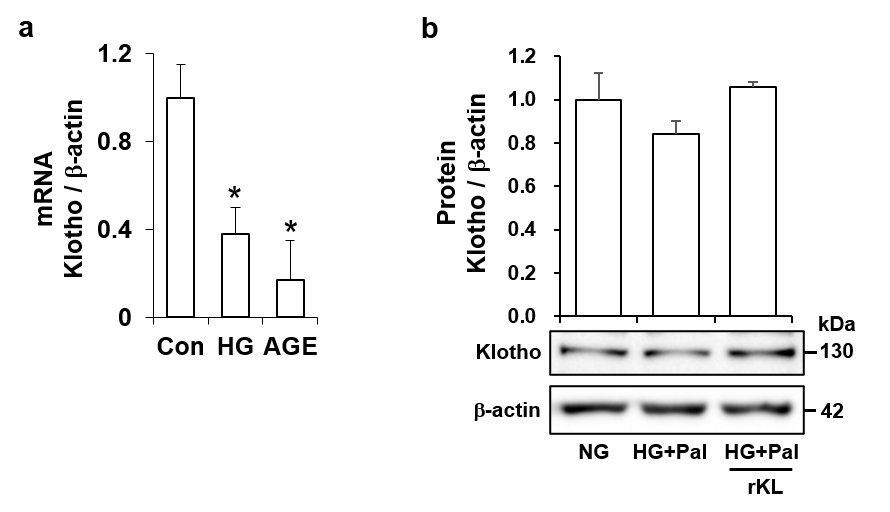
S1 Fig. Decreased klotho expression in HG- and AGE-treated mouse podocytes.** To investigate whether HG and advanced glycation end-products (AGE) could decrease expression level of klotho, mouse podocytes were treated with 30 mM HG or 100 mg/ml AGE for 24 hours. (a) klotho gene was quantified by real-time qPCR analysis and data was normalized by β-actin mRNA levels in the same sample. (b) Western blot showing decreased klotho by cotreatment of HG and Palmitate. Data is presented as the mean ± SD. *P < 0.05 versus control (CTL).
