# Supplementary material for: Protective effects of klotho on palmitate-induced podocyte injury in diabetic nephropathy
Source: PLoS One. 2021 Apr 23;16(4):e0250666. doi: 10.1371/journal.pone.0250666 (PMC8064606; doi:10.1371/journal.pone.0250666)
Supplement: Attachment — Submitted filename: Klohto_Response to Revieweres.doc [file pone.0250666.s004.doc]

Dear editor,

We would like to thank you and reviewer #1 for taking the time and effort to review our manuscript. Many of the valuable and constructive comments raised by reviewer #1 were appreciated. Based these comments, we have carefully revised our manuscript as described in the following point-by-point responses. We thank reviewer #1 for his thoughtful comments, which improved our manuscript.

**Reviewer #1**

The current study "Protective effects of klotho on palmitate-induced podocyte injury in diabetic nephropathy" made significant improvement compared with the previous version. Here are some more minor concerns to be addressed:

1. Full length of caspase-3 should be shown together with cleaved caspase-3 in western blot.

**Answer)** Thank you for your comment. As suggested, we showed cleaved caspase-3 and caspase 3 in Fig 3c.

2. TUNEL staining should show nuclear staining in figure 3.

**Answer)** Thank you for your comment. For Apo-BrdU TUNEL assays, podocytes were counter stained with propidium iodide (PI) and the Br-dUTP/TdT enzyme. Apoptotic cells showed green fluorescence, whereas red fluorescence was an indication of viable cells. We added this information in the figure legend (P13, lines 296~298). This data is shown in Fig. 3b.

3. The cause-effect relationship of FoxO3a phosphorylation and its nuclear translocation/transcriptional function is not well supported by the reference, which analyzes FoxO1. Phosphorylation and function may be two separate events. The authors should find a correct supporting reference or make a more accurate conclusion.

**Answer)** Thank you for your comment. As suggested, we find other supporting references and added the description in the discussion (P20, lines 445~459). “The FOXO transcription factor is an important regulator of longevity and cancer by regulating target genes associated with cellular differentiation, oxidative stress resistance and nutrient shortage [41]. Phosphorylation modification regulates FOXO3a activity through a cytoplasmic-nuclear shuttle mechanism. The PI3K-AKT signaling pathway regulates FOXO3a activity through phosphorylation at three conserved residues (Thr 32, Ser 253, and Ser 315). This phosphorylation excludes FOXO3a from the nucleus and induces binding to the14-3-3 nuclear export proteins, promoting its cytoplasmic accumulation and inhibiting the transactivating activity of FOXO3a [42]. Yamamoto *et al*. demonstrated that klotho reduces FOXO phosphorylation and promotes its nuclear translocation [4]. Lim *et al*. showed klotho’s protective role during tacrolimus-induced oxidative stress via FOXO3a-mediated MnSOD expression [43]. In the present study, we found that klotho decreases palmitate-induced FOXO3a phosphorylation resulting in its nuclear translocation and enhances antioxidant expression, including MnSOD. Taken together, this data suggests that klotho protects podocyte dysfunction against palmitate, which might be dependent on the FOXO3a-mediating antioxidant defense system.”

4. Many groups in the original images of western blot are not statistically analyzed. To reduce confusion, the authors can also analyze these groups, even though they do not contribute much to the conclusion.

**Answer)** Thank you for your comment. As recommended, each group was analyzed and compared in western blots.

5. Judging from Figure 5, the overall expression decrease of FoxO3a is more pronounced in the palmitate group than the phosphorylation increase. The authors should address and explain it.

**Answer)** Thank you for your comment. We wanted to investigate that palmitate-induced FOXO3a phosphorylation could be inhibited by recombinant klotho in podocytes. Thus we evaluated expression levels of phosphorylated FOXO3a compared to FOXO3a and found that palmitate increased phosphorylated FOXO3a expression, whereas recombinant klotho reduced it. We changed Figure 5b style to be clearer.
